# Monoclonal antibodies targeting IL-5/5R or IL-4/13 pathways in eosinophilic myocarditis: a single-centre experience and systematic literature review

**DOI:** 10.1093/ehjcvp/pvag025

**Published:** 2026-04-21

**Authors:** Andrea S Giordani, Caterina Menghi, Anna Baritussio, Federico Scognamiglio, Cristina Vicenzetto, Federica Davanzo, Luca Iorio, Renzo Marcolongo, Monica De Gaspari, Stefania Rizzo, Cristina Basso, Roberto Padoan, Alida L P Caforio

**Affiliations:** Cardiology and Cardioimmunology Laboratory, Department of Cardiac Thoracic Vascular Sciences and Public Health, University of Padova, Via Giustiniani 2, Padova 35128, Italy; Cardiology and Cardioimmunology Laboratory, Department of Cardiac Thoracic Vascular Sciences and Public Health, University of Padova, Via Giustiniani 2, Padova 35128, Italy; Cardiology and Cardioimmunology Laboratory, Department of Cardiac Thoracic Vascular Sciences and Public Health, University of Padova, Via Giustiniani 2, Padova 35128, Italy; Cardiology and Cardioimmunology Laboratory, Department of Cardiac Thoracic Vascular Sciences and Public Health, University of Padova, Via Giustiniani 2, Padova 35128, Italy; Cardiology and Cardioimmunology Laboratory, Department of Cardiac Thoracic Vascular Sciences and Public Health, University of Padova, Via Giustiniani 2, Padova 35128, Italy; Rheumatology, Department of Medicine, University of Padova, Via Giustiniani 2, Padova 35128, Italy; Rheumatology, Department of Medicine, University of Padova, Via Giustiniani 2, Padova 35128, Italy; Cardiology and Cardioimmunology Laboratory, Department of Cardiac Thoracic Vascular Sciences and Public Health, University of Padova, Via Giustiniani 2, Padova 35128, Italy; Cardiovascular Pathology, Department of Cardiac Thoracic Vascular Sciences and Public Health, University of Padova, Via Giustiniani 2, Padova 35128, Italy; Cardiovascular Pathology, Department of Cardiac Thoracic Vascular Sciences and Public Health, University of Padova, Via Giustiniani 2, Padova 35128, Italy; Cardiovascular Pathology, Department of Cardiac Thoracic Vascular Sciences and Public Health, University of Padova, Via Giustiniani 2, Padova 35128, Italy; Rheumatology, Department of Medicine, University of Padova, Via Giustiniani 2, Padova 35128, Italy; Cardiology and Cardioimmunology Laboratory, Department of Cardiac Thoracic Vascular Sciences and Public Health, University of Padova, Via Giustiniani 2, Padova 35128, Italy

**Keywords:** Eosinophilic myocarditis, Monoclonal antibodies, Eosinophilic granulomatosis with polyangiitis, Hypereosinophilic syndrome

## Abstract

**Aims:**

Eosinophilic myocarditis (EM) is a rare inflammatory heart disease often associated with eosinophilic granulomatosis with polyangiitis or hypereosinophilic syndrome. While anti–IL-5/5R and anti–IL-4/13 monoclonal antibodies (mAbs) efficacy in systemic eosinophilic diseases is established, data on EM are lacking. We aimed to (1) characterize a single-centre cohort of EM patients treated with mepolizumab, benralizumab, or dupilumab in combination with glucocorticoids and/or immunosuppressants; (2) systematically review published cases, comparing them with a contemporary cohort; and (3) evaluate myocardial response and safety of mAbs in EM, in comparison with a historical cohort treated without mAbs at our centre.

**Methods and results:**

Thirty-seven EM patients were included (19 from a contemporary cohort, 18 from the literature; 51% male; median age 47 years). Biologic treatments were mepolizumab (81%), benralizumab (14%), and dupilumab (5%). Median time to mAb initiation was 2.5 months; treatment duration 24 months. No EM relapses, deaths, or heart transplantations occurred. Glucocorticoids were tapered and withdrawn in 89% of cases, with no mAb discontinuations due to adverse events. In the contemporary cohort, mAb therapy was associated with improved LVEF (47%–55%, *P* = 0.004), TnI normalization (95%–12%, *P* < 0.001), and eosinophil reduction (95%–11%, *P* < 0.001). Compared with EM patients managed with conventional immunosuppressants alone, the mAb group had no myocarditis relapses (0% vs. 25%) and lower follow-up eosinophil counts (0.04 × 10^9^/L vs. 0.85 × 10^9^/L).

**Conclusion:**

In EM within eosinophilic disease, anti–IL-5/5R and anti–IL-4/13 mAbs showed steroid-sparing effects and favourable safety, suggesting potential benefit for disease control.

## Graphical Abstract

**Graphical Abstract:**
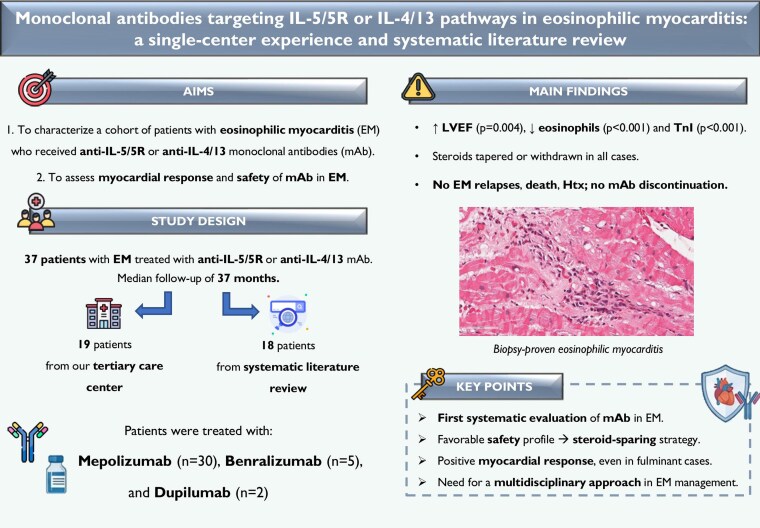

## Introduction

Eosinophilic myocarditis (EM) is a rare, potentially life-threatening inflammatory heart disease marked by eosinophilic infiltration of the myocardium.^1^ It may develop in association with a range of underlying conditions, including allergic or drug-induced reactions, infections, malignancies, systemic eosinophilic disorders such as hypereosinophilic syndromes (HES) and eosinophilic granulomatosis with polyangiitis (EGPA), or as an organ-specific immune-mediated condition.^2,3^ Clinical presentations are highly variable, ranging from asymptomatic forms to fulminant cases.^4^ In two systematic reviews, short-term mortality was high, ranging from 22% to 55%.^2,5^ Endomyocardial biopsy (EMB) is the diagnostic histological gold standard, while cardiac magnetic resonance (CMR) supports a clinically-suspected diagnosis.^6^ Immunosuppressive therapy remains the first-line treatment to prevent disease progression towards advanced stages, including endomyocardial fibrosis (Loeffler’s endocarditis).^7,8^ However, current immunosuppressive regimens, including glucocorticoids (GC) and conventional agents such as azathioprine, methotrexate, and cyclophosphamide, are non-targeted and frequently associated with significant adverse effects. This highlights the urgent need for targeted therapies that provide clinical benefit while minimizing treatment-related toxicity. Anti-interleukin-5/5R (IL-5/5R) monoclonal antibodies (mAbs), such as mepolizumab and benralizumab, and anti-interleukin-4/13 (IL-4/13), such as dupilumab, have emerged as promising candidates due to their ability to specifically target eosinophilic inflammation and improve disease control with glucocorticoid-sparing effects in EGPA and other eosinophil-driven conditions.^9,10^ Although approved for HES, EGPA, and other eosinophilic or type 2-mediated diseases, their use remains ‘off-label’ for myocardial involvement in eosinophilic systemic disease, as in EM, or when EM presents as an organ-specific immune-mediated condition.

Our aims are (1) to characterize the clinical features of a single-centre cohort of patients with EM treated with anti–IL-5/5R or anti–IL-4/13 mAbs in combination with glucocorticoids and/or immunosuppressants at our centre; (2) to perform a systematic review of the literature on EM patients treated with anti-IL-5/5R and anti-IL-4/13 mAb and compare them with a contemporary cohort; (3) to evaluate the myocardial response and safety of anti-IL-5/5R and IL-4/13 therapies in patients with EM, including comparisons with an historical cohort of patients who did not receive mAb treatment at our centre.

## Methods

Overall, this study included a total of 37 patients, of whom 19 derived from a single-centre retrospective cohort (contemporary cohort) and 18 additional cases identified through a systematic review of the literature. Patients from the contemporary cohort had EM in the context of eosinophilic diseases treated with anti-IL-5/5R or anti-IL-4/13 mAbs; the literature-derived population consisted of individual case reports or small case series describing the use of mAbs targeting IL-5/5R or IL-4/13 pathways in EM.

### Contemporary cohort experience

This retrospective analysis included 19 patients with EM in the context of eosinophilic diseases treated with anti-IL-5/5R or anti-IL-4/13 mAbs (see Supplementary material online, *Tables S1* and *S2*), followed at the Cardioimmunology and Rheumatology Vasculitis Outpatient Clinics from 2013 to 2024. Patients were followed longitudinally from the time of diagnosis, with an initial evaluation at 3 months, followed by further assessments at 6–12 months, according to disease activity, including both cardiology and rheumatology evaluations, with follow-up ongoing for all patients. For all study participants, clinical, electrocardiographic, imaging, and laboratory data were collected at diagnosis and at each follow-up outpatient visit.

Eosinophilic myocarditis was diagnosed according to the European Society of Cardiology (ESC) 2013 Position Statement and in line with the 2025 guidelines on inflammatory diseases of the myocardium.^6,11^ Myocarditis relapse was defined according to the same criteria; if an EMB was not performed at the time of relapse, the criteria for clinically suspected (CS) myocarditis had to be fulfilled. EMB was performed as clinically indicated, following current recommendations.^12^ In cases without histological confirmation, EM was CS when (1) fulfilling established criteria for CS myocarditis according to the 2013 ESC Position Statement^6,13^ in the context of eosinophilic extracardiac disease (i.e. EGPA/HES) and when (2) EMB, performed during ongoing steroid therapy, revealed lymphocytic/polymorphic myocardial infiltration, nonetheless satisfying the diagnostic criteria for CS myocarditis in the context of eosinophilic extracardiac disease.^14^ Detailed diagnostic criteria for EM are provided in the Supplementary Material (see Supplementary material online, *Tables S3* and *S4*). Patients were classified as EGPA, according to the 2022 American College of Rheumatology/European Alliance of Associations for Rheumatology classification criteria^15^ or HES according to the 2011 consensus conference of ICOG-EO.^16^

Peripheral eosinophilia was defined as an absolute eosinophil count greater than 0.5 × 10^9^/L. High-sensitivity cardiac troponin I levels were considered elevated if above 12 ng/L in women and 20 ng/L in men. Anti-heart antibodies (AHA) were assessed as previously described.^17^

Detailed characteristics and selection criteria of the historical non–mAb-treated cohort are provided in the Supplementary Methods.

The study complies with the Declaration of Helsinki and was approved by the local Ethics Committee (protocol number 0027841).

### Contemporary cohort treatment protocols

Monoclonal antibodies targeting IL-5/IL-5R or IL-4R were not initiated solely based on a cardiac indication, as these agents are currently off-label for EM. Accordingly, in the contemporary cohort, mAbs were introduced exclusively based on approved indications to treat the systemic disease. The choice of anti-IL-5/5R or anti-IL-4/13 mAbs was individualized by the multidisciplinary team based on the predominant clinical indication at treatment start, prior therapies and response, and local eligibility/reimbursement policies. Benralizumab 30 mg every 4 weeks for 3 doses then every 8 weeks, dupilumab 300 mg and mepolizumab 100 mg were generally prescribed for severe eosinophilic asthma (SEA) and/or CRSwNP, whereas mepolizumab 300 mg was more frequently chosen when systemic eosinophilic manifestations were a primary therapeutic target, as induction or maintenance of remission, according with recent recommendations.^18^

At diagnosis, all patients received systemic GC as induction therapy aimed at achieving remission of systemic eosinophilic disease and controlling myocardial inflammation. Initial regimens consisted of either intravenous methylprednisolone pulses followed by oral prednisone or oral prednisone alone, according to clinical severity. Glucocorticoids tapering was individualized and guided by clinical course, cardiac imaging, circulating biomarkers, and extracardiac disease activity.

Adverse events (AEs) were not prospectively or systematically collected, and no pre-specified AE list or monitoring schedule was applied. Safety information was obtained retrospectively from available clinical records. For the entire cohort, we specifically screened records for severe AEs, defined as events leading to death, disability, hospitalization, and/or prolongation of existing hospitalization. For a subset of patients with more detailed follow-up documentation, non-severe events were also extracted and are reported descriptively.

### Systematic review of the literature

A systematic literature search was performed using PubMed (MEDLINE) and EMBASE from inception of each database to 15 January 2025. This systematic review was performed according to the Preferred Reporting Items for Systematic Reviews and Meta Analyses (PRISMA) recommendations.^19^ The search strategy included the following keywords: [myocarditis(tiab)] AND (mepolizumab OR benralizumab OR dupilumab). Then, reference lists of eligible case reports or case series were manually screened to identify any additional pertinent case report.

**Figure 1:**
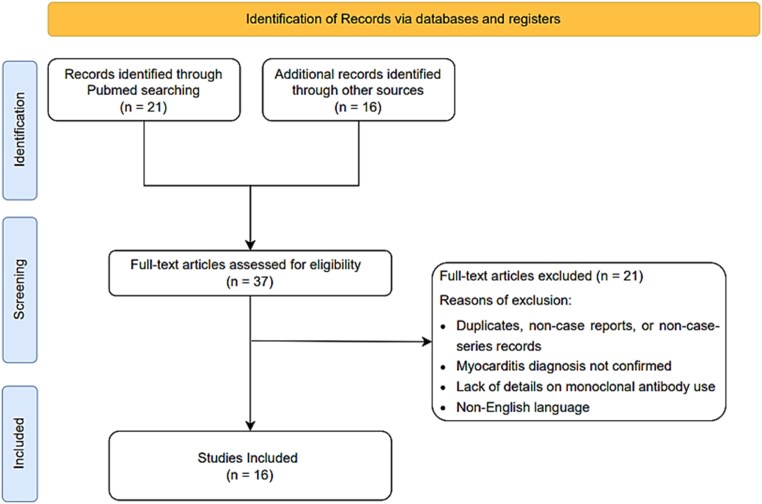
PRISMA flow diagram illustrating the systematic review process. The diagram outlines the identification of records, full-text assessment, and reasons for exclusion based on predefined eligibility criteria, resulting in 16 studies included in the final systematic review.

The inclusion criteria were a diagnosis of EMB-proven or CS EM, and the use of a mAb targeting IL-5/5R or IL-4/13. Exclusion criteria were: (1) lack of sufficient criteria for myocarditis diagnosis; (2) lack of details on mAb use. Data were extracted from each case report based on the following categories: demographic characteristics, clinical features of myocarditis and therapeutic strategies. Data were entered into Microsoft Excel (Microsoft Corp., Seattle, WA) using a standardized extraction template. Two authors (A.S.G. and C.M.) independently extracted and recorded data. Disagreements were resolved through discussion; if consensus could not be reached, a third author (F. D.) was designated to make the final decision. The flow diagram outlining the study selection process is shown in *Figure 1*. All the included cases (*n* = 18) were rated as being of good quality as assessed by the Murad tool (score ≥6, Supplementary material online, *Table S5*).^20^

### Statistical analysis

Summary statistics of clinical and instrumental variables were expressed as median and interquartile range (Q1-Q3), or absolute number and percentage, as appropriate. Continuous variables were compared using Mann–Whitney U test; categorical variables were compared using Pearson’s Chi-square tests and Fisher’s exact tests. To account for multiple testing, *P*-values were adjusted using the Benjamini–Hochberg false discovery rate (FDR) procedure. Variables at the time of diagnosis and at last follow-up were compared using paired data analysis, applying McNemar’s test for categorical variables and the Wilcoxon signed-rank test for continuous variables. Statistical significance was based on a two-sided *P*-value threshold of < 0.05. The analyses were performed using the Jamovi software (version 2.3).

## Results

### Baseline patients’ characteristics

A total of 37 patients with EM treated with anti-IL-5/5R or anti-IL-4/13 agents for concomitant extra-cardiac eosinophilic disorders were included [51% male; median age at diagnosis 47 years, IQR (34–60)]; 19 from the contemporary cohort (see Supplementary material online, *Table S6*) and 18 identified through a systematic literature review from 16 different studies.^21–36^ Baseline clinical, diagnostic, and therapeutic characteristics are detailed in *Table 1*. The contemporary cohort and the literature-derived population were broadly comparable, with some differences. In the contemporary cohort, 6 patients (46%) were AHA positive; no corresponding data were available for the literature. An acute coronary syndrome (ACS)–like presentation at EM diagnosis was more frequent in the contemporary cohort (68% vs. 17%, *P* = 0.004), whereas heart failure (HF) presentation (43% vs. 68%, *P* = 0.004) and NYHA class II–III at diagnosis (32% vs. 89%, *P* = 0.007) predominated in the literature subgroup. GC were administered as first-line therapy in 95% of patients at the time of EM diagnosis, with a median oral dosage of 75 mg/daily and preceded by methylprednisolone pulses in 54% of the cohort (*n* = 19); additional immunosuppressants (e.g. rituximab) were used more frequently in the contemporary cohort (63% vs. 28%, *P* = 0.031), primarily for the systemic syndromes’ treatment (see Supplementary material online, *Table S7*).

**Table 1:** Baseline features of the study cohort

Characteristic	N	Total *n* = 37	Contemporary cohort *n* = 19	Literature *n* = 18	*P*-value^b^
**Male sex**	37	19 (51%)	11 (58%)	8 (44%)	0.41
**Age** (years)	37	47 (34, 60)	47 (38, 58)	48 (24, 61)	0.64
**Systemic eosinophilic diseases**	37				0.22
EGPA		24 (65%)	15 (79%)	9 (50%)	
HES		6 (16%)	2 (11%)	4 (22%)	
Eosinophilic Asthma		5 (13%)	2 (11%)	3 (17%)	
**Cardiac symptoms at diagnosis**	35	32 (86%)	14 (82%)	18 (100%)	0.10
**Allergy**	23	16 (70%)	13 (68%)	3 (75%)	>0.99
**Asthma**	37	31 (84%)	18 (95%)	13 (72%)	0.090
**BVASv3**	22	15.50 (12.25, 20,75)	14.0 (12.0, 16.5)	20.0 (16.5, 22.0)	0.12
**Multisystem involvement**	24				
Constitutional symptoms	24	14 (58%)	9 (53%)	5 (71%)	0.65
Cutaneous symptoms	24	7 (29%)	4 (24%)	3 (43%)	0.37
ENT symptoms	24	17 (71%)	13 (76%)	4 (57%)	0.37
Pulmonary symptoms	24	24 (100%)	17 (100%)	7 (100%)	
Digestive symptoms	24	4 (17%)	1 (5.9%)	3 (43%)	0.059
Renal symptoms	24	1 (4%)	0	1 (14%)	0.29
Neurological symptoms	24	8 (33%)	4 (24%)	4 (57%)	0.17
**EM clinical presentation**	37				**0**.**004**
Heart failure		18 (49%)	5 (26%)	13 (72%)	
Acute coronary syndrome-like		16 (43%)	13 (68%)	3 (17%)	
Asymptomatic cardiac involvement		3 (8%)	1 (5.3%)	2 (11%)	
**NYHA II-III at diagnosis**	28	15 (53%)	6 (32%)	9 (90%)	**0**.**007**
**Fulminant EM presentation**	37	7 (19%)	2 (11%)	5 (28%)	0.23
**Biomarkers**					
TnI elevation	37	30 (81%)	18 (95%)	12 (71%)	0.081
TnI level (ng/L)	26	3261 (864, 5114)	3473 (806, 5000)	2231 (1,046, 12,590)	0.94
CRP elevation	37	22 (59%)	14 (74%)	8 (47%)	0.10
CRP level (mg/L)	22	36 (14, 93)	28 (12, 108)	44 (21, 78)	0.82
NT-proBNP elevation	32	23 (70%)	13 (87%)	10 (59%)	0.070
NT-proBNP level (pg/mL)	19	1557 (725, 5568)	1180 (528, 5790)	1654 (1,418, 4041)	0.4
Eosinophils elevation	37	36 (97%)	18 (95%)	18 (100%)	>0.99
Eosinophil count (×10^9^/L)	33	5 (2, 10)	8 (4, 12)	3 (2, 8)	0.20
**Autoantibodies profile at diagnosis**					
AHA positivity	13	6 (46%)	6 (46%)		>0.99
AIDA positivity	13	4 (31%)	4 (31%)		>0.99
ANCA positivity	23	1 (4%)	0	1 (12%)	0.35
**Abnormal ECG** ^a^	22	14 (63%)	12 (63%)	2 (67%)	>0.99
**Echocardiography**					
LVEF < 50%	37	27 (73%)	14 (74%)	13 (72%)	>0.99
LVEF (%)	20	47 (41, 55)	48 (43, 57)	41 (40, 48)	0.53
LVEDVi (mL/m^2^)	16	68 (54, 77)	68 (54, 77)		
TAPSE (mm)	12	22 (18, 25)	22 (18, 25)		>0.99
FAC (%)	16	42 (36, 48)	42 (36, 48)		
Moderate/severe mitral regurgitation	18	6 (33%)	6 (33%)		>0.99
Pericardial effusion	37	13 (35%)	6 (32%)	7 (39%)	0.64
Endocavitary thrombus	37	8 (22%)	6 (32%)	2 (11%)	0.23
**CMR**	37	32 (86%)	17 (89%)	15 (83%)	0.66
Oedema	15	11 (73%)	11 (73%)	0	>0.99
LGE	34	32 (94%)	17 (100%)	15 (88%)	0.48
LGE pattern	32				>0.99
Subendocardial (ischaemic pattern)		29 (91%)	14 (82%)	15 (100%)	
Epicardial/intramyocardial spots		3 (9%)	3 (18%)	0 (0%)	
**Endomyocardial biopsy at diagnosis**	37	19 (51%)	7 (37%)	12 (67%)	0.07
Histological type	16				0.0008
Eosinophilic		12 (75%)	2 (33%)	10 (100%)	
Lymphocytic		3 (19%)	3 (50%)	0	
Polymorph		1 (6%)	1 (17%)	0	
PCR on EMB	19	7 (37%)	7 (100%)	0	<0.001
Negative PCR results		7(100%)	7 (100%)		
**Treatment**					
**Type of mAb**	37				0.826
Mepolizumab		30 (81%)	16 (84%)	14 (78%)	
Benralizumab		5 (14%)	2 (11%)	3 (17%)	
Dupilumab		2 (5%)	1 (5%)	1 (5%)	
**Time from myocarditis onset to mAb therapy initiation** (months)	36	2.5 (1.0, 16,0)	12 (2, 48)	1 (0, 3)	**0**.**005**
**GC use at diagnosis**	35	33 (94%)	18 (95%)	15 (94%)	>0.99
**MPN pulses at diagnosis**	35	19 (54%)	9 (47%)	10 (62%)	0.50
**GC dosage at diagnosis**	34	75 (50, 75)	75 (55, 75)	60 (45, 75)	0.27
**IS or other immunomodulatory treatments at diagnosis**	37	17 (46%)	12 (63%)	5 (28%)	**0**.**031**

Data are presented as median (Q1–Q3) or N (%). Statistically significant values in bold (*P* < 0.05).

AHA, anti-heart autoantibodies; AIDA, anti-intercalated disk autoantibodies; ANCA, anti-neutrophil cytoplasmic antibodies; BNP, brain natriuretic peptide; BVAS, Birmingham Vasculitis Activity Score; CMR, cardiovascular magnetic resonance; CRP, C-reactive protein; cTn, cardiac troponin; EGPA, eosinophilic granulomatosis with polyangiitis; EMB, endomyocardial biopsy; ENT, ear-nose-throat; FAC, fractional area change; GC, glucocorticoid; HES, hypereosinophilic Syndrome; IS, immunosuppression; LGE, late gadolinium enhancement; LVEDVi, indexed left ventricular end-diastolic volume; LVEF, left ventricular ejection fraction; mAb, monoclonal antibody; MPN: methylprednisolone; NYHA, New York Heart Association; PCR, polymerase chain reaction; RF, rheumatoid factor; RVED, right ventricular end-diastolic; TAPSE, tricuspid annular plane systolic excursion.

^a^Abnormal ECG was defined as the presence of any repolarization (ST-T abnormalities), depolarization (pathological Q waves, low QRS voltage), or conduction disturbances (AV or bundle branch block).

^b^Benjamini and Hochberg correction for multiple testing.

Mepolizumab was the most frequently prescribed mAb (81%, *n* = 30), followed by benralizumab (14%, *n* = 5) and dupilumab (5%, *n* = 2). All patients received mAb monotherapy without any combination or switching. Dosage regimens were as follows: dupilumab 300 mg every 2 weeks; mepolizumab 300 mg every 4 weeks (100 mg in two patients); and benralizumab 30 mg every 4 weeks for the first three doses, then every 8 weeks. Regarding clinical indications at initiation, dupilumab was started for uncontrolled SEA (*n* = 1); benralizumab was administered for uncontrolled SEA (*n* = 1) and as a steroid-sparing maintenance therapy in EGPA (*n* = 1); Mepolizumab was primarily utilized for remission maintenance (*n* = 6) or induction (*n* = 6) of EGPA/HES, and occasionally for uncontrolled SEA and/or CRSwNP (see Supplementary material online, *Table S6*).

In the contemporary cohort, mAb were introduced relatively late in the disease course when compared with those published in the literature (median 12 vs. 1 month, *P* = 0.005). This reflects our treatment strategy, in which remission of the systemic eosinophilic disorder was first induced with GC, sometimes combined with immunosuppressants/immunomodulant therapies, and biologics were subsequently used as maintenance therapy to consolidate remission and sustain control. By contrast, in four cases reported in the literature, anti–IL-5 mAbs (mepolizumab) were administered upfront as part of the induction regimen alongside GC, with good tolerability and no relapses.^27,28,31,35^

Most patients (35/37) received mAbs according to European Medical Agency (EMA)-approved indications, mainly as maintenance therapy for EGPA/HES; exceptions included two literature cases treated ‘off-label’ for fulminant EM with haemodynamic compromise.^28,35^ In the contemporary cohort, two patients with virus-negative EM and refractory HF initially required standard immunosuppressive therapy. After myocardial recovery and a subsequent diagnosis of EGPA and SEA, mepolizumab and dupilumab were respectively introduced, in line with approved indications.^18,37,38^

### Patients’ characteristics at follow-up

At follow-up, no cases of EM relapse, death, or heart transplantation were reported across the entire cohort. GC therapy was successfully tapered and discontinued in 89% of patients, without need for reintroduction or escalation of additional immunosuppressive agents. No patient discontinued mAb therapy. No biologic-related severe AEs (death, disability, hospitalization) were identified in available records. Among the subset with detailed safety documentation (13/19), three patients reported mild events (somnolence, nausea, arthralgia/myalgia), none requiring discontinuation. In the contemporary cohort of 19 patients who were longitudinally followed for a median of 37 months (Q1–Q3: 26–65) (*Table 2*), long-term myocardial benefit and safety of mAb therapy were observed. A stable and significant improvement in LVEF was noted from diagnosis to last follow-up (median 47% at baseline vs. 56% at follow-up; *P* = 0.004), accompanied by normalization of troponin I levels (95% of patients had elevated troponin at diagnosis, whereas only 2 patients showed persistent release at last follow-up; *P* < 0.001) and peripheral eosinophil counts (95% had eosinophilia at baseline, compared with 11% at last follow-up; *P* < 0.001).

**Table 2:** Follow-up features of the 19 myocarditis patients treated with anti-IL-5/5R or anti-IL-4/13 from the contemporary cohort

Characteristic	N	Padova *n* = 19
**Follow-up duration (months)**	19	37 (26, 65)
**NYHA II-III at follow-up**	19	3 (16%)
**Biomarkers**		
TnI elevation	17	2 (12%)
PCR elevation	19	0
NTproBNP elevation	18	9 (50%)
Eosinophils elevation	19	2 (11%)
**Abnormal ECG**	16	8 (50%)
**Echocardiography**		
LVEF <50%	18	3 (17%)
LVEF (%)	19	56 (52, 61)
VTDi (mL/m^2^)	17	63 (52, 67)
TAPSE (mm)	13	20 (18, 26)
FAC (%)	15	50 (42, 56)
ATD (mm^2^)	15	20.0 (15.0, 21.5)
**CMR**	19	10 (53%)
Oedema on CMR	10	0
LGE on CMR	10	9 (90%)
LGE pattern	9	
Subendocardial (ischaemic pattern)		6 (67%)
Epicardial/intramyocardial spots		3 (33%)
Reduced number of LGE-positive segments	9	5 (56%)
**Therapy at follow-up**		2
mAb therapy withdrawn	19	0
Ongoing GC therapy	19	2 (11%)
Ongoing IS/other immunomodulatory therapy	19	0
**Total duration of mAb therapy**, months	19	24 (15, 33)
**Total duration of GC therapy**, months	17	15 (10, 35)
**Duration of mAb therapy without concomitant GC or IS**, months	19	17 (6, 25)

Values are median (Q1, Q3) or n (%).

CMR, cardiac magnetic resonance; FAC, fractional area change; GC, glucocorticoid; IS, immunosuppression; LGE, late gadolinium enhancement; LVEF, left ventricular ejection fraction; mAb, monoclonal antibody; PCR, polymerase chain reaction; TAPSE, tricuspid annular plane systolic excursion.

**Figure 2:**
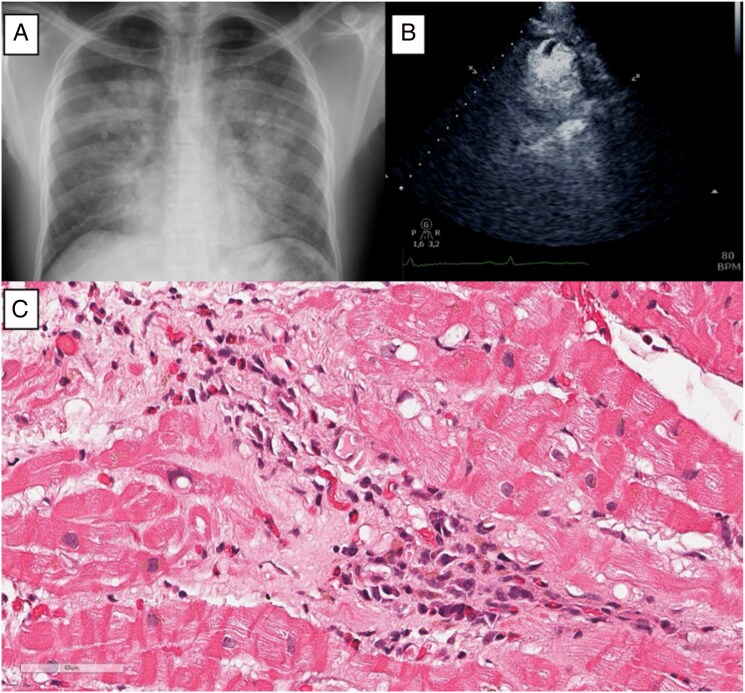
Diagnostic findings of a patient from the contemporary cohort. An 80-year-old patient with long-standing history of asthma and no previous cardiological history presented to the Emergency department because of worsening dyspnoea; clinical scenario and chest X ray findings (*A*) were consistent with acute pulmonary oedema. Peripheral eosinophils were markedly elevated (8.05 × 10^9^/L). Transthoracic echocardiography showed reduction in left ventricular ejection fraction (40%) and endocavitary thrombus (*B*, apical parasternal short axis view following administration of echographic contrast agent). Hs-Troponin I was abnormal (360 ng/L), but invasive coronary angiography revealed absence of significant coronary artery disease. Endomyocardial biopsy (*C*) was performed, showing eosinophilic virus-negative myocarditis with associated thrombosis (Loeffler’s endocarditis). The patient was first treated with high dose glucocorticoids with rapid symptoms resolution and left ventricular function normalization. Mepolizumab was initiated, leading to rapid steroid treatment tapering, with excellent tolerability and safety profile.

In a subgroup analysis comparing patients with biopsy-proven eosinophilic myocarditis (BP-EM, *n* = 11, see *Figure 2*) with the remaining cohort (CS-EM, *n* = 26), which included both BP cases without detectable eosinophilic infiltration and CS eosinophilic myocarditis, we found a higher prevalence of HF at presentation and more frequently normal troponin levels in the BP group, while the interval to mAb initiation was shorter in this subgroup (see Supplementary Results and Supplementary material online, *Tables S8* and *S9*).

### Comparison of EM patients treated with vs. without mAbs

Finally, we identified a historical cohort of 12 patients with biopsy proven EM followed at our centre who did not receive mAb therapy (see Supplementary material online, *Table S10*). We compared these 12 patients with the overall study cohort of EM patients treated with mAbs. Among the 12 patients not treated with mAbs, glucocorticoid alone was used in 2 cases, while glucocorticoid was combined with methotrexate in 3, azathioprine in 3 and cyclosporine in 1.^39,40^ Baseline and follow-up clinical characteristic were broadly similar; however, non–mAb-treated patients had lower prevalence of asthma (8% vs. 84%, *P* < 0.001) and higher absolute eosinophil counts at follow-up (median 0.85 [0.74–0.95] × 10^9^/L vs. 0.04 [0.03–0.08] × 10^9^/L; *P* = 0.043). Moreover, the pattern of late gadolinium enhancement (LGE) on CMR was more frequently epicardial/intramyocardial in the non–mAb-treated group (57% vs. 9%, *P* = 0.003). Importantly, myocarditis relapses were observed only in patients not receiving mAb therapy (25% vs. 0%; *P* = 0.012).

## Discussion

This is the first study to systematically characterize a cohort of patients with EM treated with anti–IL-5/5R and anti–IL-4/13 mAbs for concomitant extracardiac eosinophilic diseases, primarily EGPA and HES. Our findings demonstrate that: (1) mAbs targeting eosinophilic pathways were well tolerated, with no treatment discontinuations due to AEs and successful glucocorticoids withdrawal achieved in nearly all patients; (2) beyond their established efficacy in extracardiac eosinophilic manifestations, we provide the first preliminary evidence suggesting a potential myocardial benefit of these agents as maintenance therapy for myocarditis, as indicated by the absence of EM relapse, death, or heart transplantation, alongside improvements in LVEF and normalization of troponin I levels from diagnosis to last follow-up. These observations extend real-world data in EGPA, where IL-5/5R blockade has been shown to induce sustained remission and marked steroid-sparing effects.^41,42^

### mAbs targeting IL-5/5R and IL-4/13 in eosinophilic myocarditis: evidence of myocardial benefit beyond extracardiac eosinophilic indications

To date, mepolizumab 100 mg/4 weeks and benralizumab 30 mg/8 weeks are approved by European Medicines Agency (EMA) for severe eosinophilic asthma, while mepolizumab 300 mg/4 weeks is approved for EGPA and HES. Dupilumab is approved for multiple eosinophilic-mediated diseases, including atopic dermatitis, asthma, CRwNP, and eosinophilic esophagitis. However, no evidence-based guidelines exist for EM, and high-dose glucocorticoids remain the mainstay of treatment, along with conventional immunosuppression regimens (such as azathioprine, MMF, etc.) as per biopsy-proven virus-negative myocarditis according to the TIMIC protocol.^2,37^

In relapsing or refractory EGPA, a randomized controlled trial showed that mepolizumab added to standard therapy significantly reduced relapse rates and allowed greater glucocorticoid sparing compared with placebo.^43^ In HES, retrospective data suggest that cardiac involvement predicts poorer response to mepolizumab; however, myocardial involvement was not rigorously defined. Notably, affected patients had a median disease duration of 4.6 years prior to therapy initiation, suggesting a possible window for early intervention.^44^ In a placebo-controlled trial of benralizumab in HES, 74% achieved clinical response. Among two patients with cardiac involvement, one with endomyocardial fibrosis was unresponsive, whereas the other, with steroid-dependent HES and acute heart failure, showed improved LVEF after early benralizumab initiation, supporting the benefit of timely treatment.^45^ Of note, in the contemporary cohort, mAbs were introduced primarily as maintenance therapy at an earlier stage of the disease than reported in the above studies.^44,45^ The earlier administration—facilitated by close multidisciplinary collaboration—may partly account for the favourable myocardial response observed, including the absence of disease relapse, death, and heart transplantation, and the improvement in LVEF over long-term follow-up. Consistently, our systematic review of single-case reports showed comparable benefits of mAbs as maintenance therapy. Notably, in four cases, mAbs were administered as induction therapy during hospitalization for severe disease manifestations,^27,28,31,35^ suggesting a potential role even as an upfront therapeutic strategy, with favourable effects on myocardial protection and long-term prognosis in EM. Importantly, myocardial improvement was observed even in fulminant EM presentations, including two patients from the contemporary cohort who required inotropic support and five from the literature.^25–27,32,35^ The therapeutic rationale for mAbs in EM lies in the targeted blockade of IL-5/5R and IL-4/13, key cytokines in eosinophil maturation, activation, and tissue recruitment, thereby reducing myocardial eosinophilic infiltration^46^; thus, early administration may help prevent progression to endomyocardial fibrosis, which is often irreversible once established.^23,35^

### Comparative analysis of conventional and targeted immunotherapies in eosinophilic myocarditis

In our study, patients with biopsy-proven EM who did not receive mAbs appeared more vulnerable to myocarditis relapse than those treated with mAbs, with relapse occurring in 25% of the non–mAb-treated group vs. none of the mAb-treated patients. Moreover, non–mAb-treated patients had higher peripheral eosinophil counts at follow-up, suggesting that persistent eosinophilia may expose patients to a greater risk of relapse, in line with observations from extracardiac eosinophilic diseases where deeper eosinophil depletion has been associated with more stable disease control, including EGPA.^40^

### Role of endomyocardial biopsy in guiding anti-IL-5/5r and IL-4/13 targeted therapies

EMB remains the gold standard for the histological diagnosis of EM, also allowing for definitive exclusion of myocardial infective agents. In our study, EMB was performed in nearly half of the overall cohort; however, histological confirmation of eosinophilic infiltration was not obtained in four cases (three with lymphocytic and one with polymorphic infiltrates), likely due to ongoing high-dose glucocorticoid therapy at the time of endomyocardial biopsy. This highlights a well-recognized limitation in EMB’s diagnostic yield when performed after steroid initiation.^14,47^ In such cases, a diagnosis of CS EM was still possible based on characteristic CMR imaging findings.^48^ Notably, although EMB was not performed in all cases, particularly when myocardial eosinophilic infiltration was strongly suspected in the context of EGPA/HES and in the absence of formal indications for biopsy,^12^ no significant differences in myocardial response were observed between BP and CS EM patients. Nevertheless, the shorter time to mAb initiation in the BP subgroup supports the concept that histological confirmation may facilitate more timely and targeted therapeutic decisions when an EMB is promptly performed.

### Safety and tolerability of anti–IL-5/5r and anti–IL-4/13 agents

Given their favourable safety profile compared with GC and conventional immunosuppressants, anti-IL-5/5R and anti-IL-4/13 mAbs should be considered early in the treatment algorithm, particularly in steroid-dependent or relapsing cases. Remarkably, in the contemporary cohort, no patient discontinued mAb therapy throughout the follow-up period, with a median treatment duration of 24 months. GC were successfully discontinued in most patients (89%), and all additional immunosuppressive or immunomodulatory agents were withdrawn. The two patients who remained on GC at last follow-up were maintained on a low-dose regimen of prednisone 2.5 and 7.5 mg daily, respectively, with a planned tapering schedule. Currently, no robust evidence or consensus exists regarding the optimal duration of mAb therapy, either in EGPA/HES or specifically in EM. Prospective studies with extended follow-up are warranted to establish criteria for safely tapering or discontinuing treatment.

### Humoral immunity in eosinophilic myocarditis: beyond eosinophilic-mediated organ damage

In the overall cohort, most patients tested negative for ANCA, supporting the notion that cardiac involvement in EGPA/HES is more commonly ANCA-negative.^3^ Beyond ANCA profiling, this is the first study to report the prevalence of AHA and AIDA in patients with EGPA or HES and BP or CS myocardial involvement: 46% and 31% of patients tested positive for AHA and AIDA, respectively. These results are in line with those previously reported in other contexts of immune-mediated myocarditis.^49^ This supports the hypothesis that, alongside eosinophil-mediated cytotoxicity, humoral immune mechanisms may contribute to myocardial injury. Indeed, the involvement of AHA and AIDA is established in other cardiac and extracardiac immune-mediated diseases, with strong prognostic value.^50,51^

### Limitations

Our study has several limitations. First, the combination of a single-centre retrospective cohort with literature-derived case reports limits the ability to draw causal inferences regarding the efficacy of mAbs in EM. Accordingly, the present findings should be regarded as hypothesis-generating and reflective of observed associations rather than definitive treatment effects. Second, we acknowledge the potential for time-related bias and treatment-sequencing confounding, as mAbs were frequently initiated following induction with GC and/or other immunosuppressants. Third, although favourable safety and outcome signals were observed, definitive conclusions regarding safety or comparative effectiveness vs. standard immunosuppressive regimens cannot be established. Safety findings should be interpreted cautiously because non-severe AEs were not systematically collected and were only available for a subset of patients.

Fourth, in literature-derived cases, coronary artery disease was not consistently excluded prior to EM diagnosis through dedicated assessment (e.g. invasive coronary angiography or cardiac CT); however, all cases met clinical, non-invasive, and/or invasive diagnostic criteria for eosinophilic myocarditis. Moreover, EMB—when performed—was not uniformly accompanied by viral genome polymerase chain reaction testing in literature cases, whereas this evaluation was systematically conducted in our single-centre cohort.

In addition, although no EM relapses were observed in the whole cohort, follow-up intensity and duration vary across literature cases, which may contribute to under-ascertainment of events in published reports.

Finally, comparisons between mAb-treated and non-mAb-treated EM patients were based on retrospective, non-matched data.

Despite these limitations, this study represents the first systematic exploration of targeted biologic therapies in EM and provides a biologically plausible and clinically relevant signal that warrants confirmation in prospective, multicentre studies with larger patients’ cohort.

## Conclusions

Anti–IL-5/5R and anti–IL-4/13 mAbs show a favourable safety profile and provide preliminary evidence of potential long-term myocardial benefit in EM patients, in addition to their well-established extracardiac efficacy in controlling the systemic manifestations of eosinophilic disorders. This targeted therapeutic approach may represent an early option in EM, allowing for GC tapering or withdrawal and reducing the need for non-targeted immunosuppression. Future studies should aim to identify predictors of myocardial response and validate these findings in larger, prospective patient cohorts.

## Supplementary Material

pvag025_Supplementary_Data

## Data Availability

The data underlying this article will be shared on reasonable request to the corresponding author.
