# Supplementary material for: Monoclonal antibodies targeting IL-5/5R or IL-4/13 pathways in eosinophilic myocarditis: a single-centre experience and systematic literature review
Source: Eur Heart J Cardiovasc Pharmacother. 2026 Apr 21;12(4):320–30. doi: 10.1093/ehjcvp/pvag025 (PMC13367243; doi:10.1093/ehjcvp/pvag025)
Supplement: pvag025_Supplementary_Data [file pvag025_supplementary_data.zip › V2_Mepolizumab_Supplementary_01.04.2026.docx]

**SUPPLEMENTARY METHODS**

**Historical cohort characterization**

The historical comparator cohort consisted of consecutive patients with biopsy-proven eosinophilic myocarditis who fulfilled the same inclusion and exclusion criteria applied to the primary monoclonal antibody–treated cohort (see Supplementary Table 1 and 2) and were diagnosed and followed at our tertiary referral center between 2010 and 2020. This time window precedes the widespread availability and routine use of monoclonal antibodies for eosinophilic disorders in clinical practice. During active follow-up, patients were managed according to the same institutional diagnostic and surveillance protocol adopted for the primary cohort, including standardized clinical assessment, laboratory testing, and multimodality cardiac imaging. These patients were subsequently lost to structured longitudinal follow-up and therefore are not currently receiving monoclonal antibody therapy, which justified their use as a historical non–mAb-treated comparator group.

**SUPPLEMENTARY RESULTS**

**Comparison between biopsy-proven and clinically suspected eosinophilic myocarditis**

A subgroup analysis compared patients with biopsy-proven eosinophilic myocarditis (EM-BP, n = 11) with the remaining cohort (EM-CS, n = 26), which included individuals with BP myocarditis without documented eosinophilic infiltration (most likely related to prior glucocorticoid exposure at the time of endomyocardial biopsy) and those diagnosed with clinically suspected eosinophilic myocarditis. Baseline and follow-up clinical, laboratory, and imaging characteristics were largely similar between the two groups, although several significant differences emerged (Supplementary Tables 7 and 8). Eosinophilic granulomatosis with polyangiitis was more frequent among patients without histological confirmation of eosinophilic infiltration (77% vs. 36%, p = 0.023), whereas heart failure at initial presentation and normal troponin I values were more common in the BP-EM group (82% vs. 35%, p = 0.044; 45% vs. 4%, p = 0.006). Time from diagnosis to initiation of monoclonal antibody therapy was shorter in patients with EM-BP (median 0 vs. 8 months, p = 0.007). No differences were observed between groups regarding myocarditis relapse, mortality, or heart transplantation during follow-up.

**Supplementary Table 1. Inclusion criteria for patients with eosinophilic myocarditis treated with anti-IL-5/5R or anti-IL-4/13 monoclonal antibodies**

| Category |  |
| --- | --- |
| Criteria related to myocarditis | **a) Biopsy-proven eosinophilic myocarditis**   - EMB-proven EM according to ESC 2013 Position Statement. - In cases where EMB was performed during ongoing steroid therapy, the presence of lymphocytic or polymorphic inflammatory infiltrates was accepted, provided that the clinical picture was consistent with CS EM in the context of systemic eosinophilic disease.   **b) Clinically suspected eosinophilic myocarditis**   - Fulfilment of clinical criteria for myocarditis according to ESC 2013 Position Statement. - Evidence of cardiac involvement compatible with EM (including CMR findings showing subendocardial LGE not confined to a coronary artery territory) without histological confirmation, provided that it occurs in the context of a documented systemic eosinophilic disease. |
| Criteria related to systemic eosinophilic disease | - Diagnosis of EGPA, HES or severe eosinophilic asthma. |
| Treatment | Use of monoclonal antibody targeting IL-5/5R (mepolizumab, benralizumab) or IL-4/13 (dupilumab). |
| Data availability / reporting | Clinical, laboratory, and therapeutic data available in medical records and study case reports/case series. |

**Legend**: ACR/EULAR: American College of Rheumatology / European Alliance of Associations for Rheumatology; CMR: Cardiac magnetic resonance; CS: Clinically suspected; EM: Eosinophilic myocarditis; EMB: Endomyocardial biopsy; EGPA: Eosinophilic granulomatosis with polyangiitis; ESC: European Society of Cardiology; GINA: Global Initiative for Asthma; HES: Hypereosinophilic syndrome; ICS: Inhaled corticosteroids; IL-4/13: Interleukin-4 / Interleukin-13; IL-5/5R: Interleukin-5 / Interleukin-5 receptor; LGE: Late gadolinium enhancement.

**Supplementary Table 2. Study Exclusion Criteria**

Patients were excluded if alternative conditions could account for the clinical, imaging, or histopathological findings, or if data were insufficient for a definitive diagnosis.

| Category | Description |
| --- | --- |
| 1. Structural heart disease | Pre-existing structural heart disease, including valvular, congenital, ischemic, or genetic cardiomyopathy. |
| 2. Takotsubo cardiomyopathy | Stress-induced (Takotsubo) cardiomyopathy. |
| 3. Other infiltrative/inflammatory diseases | Cardiac sarcoidosis, amyloidosis, or other infiltrative diseases confirmed by histology or compatible multimodality imaging findings. |
| 4. Incomplete diagnostic dataset | Missing clinical, laboratory, or imaging data precluding diagnostic confirmation according to ESC and updated Lake Louise criteria. |

**Supplementary Table 3. Diagnostic Criteria for Clinically Suspected Myocarditis**

Adapted from Caforio et al. [6]

| **Clinical presentations** |
| --- |
| - Acute chest pain, pericarditic, or pseudo-ischaemic - New-onset (days up to 3 months) or worsening of dyspnoea at rest or exercise, and/or fatigue, with or without left and/or right heart failure signs - Subacute/chronic (>3 months) or worsening of dyspnoea at rest or exercise, and/or fatigue, with or without left and/or right heart failure signs - Palpitation, and/or unexplained arrhythmia symptoms, and/or syncope, and/or aborted sudden cardiac death - Unexplained cardiogenic shock |
| **Diagnostic criteria** |
| **I. ECG/Holter/stress test features**   - Newly abnormal 12-lead ECG and/or Holter and/or stress testing, showing any of the following: - I to III degree atrioventricular block or bundle branch block - ST/T wave change (ST elevation or non-ST elevation, T-wave inversion) - Sinus arrest, ventricular tachycardia/fibrillation, asystole, atrial fibrillation - Reduced R-wave height, intraventricular conduction delay (widened QRS complex), abnormal Q waves, low voltage, frequent premature beats, supraventricular tachycardia   **II. Myocardiocytolysis markers**   - Elevated TnT/TnI   **III. Functional and structural abnormalities on cardiac imaging (echo/angio/CMR)**   - New, otherwise unexplained LV and/or RV structure and function abnormality (including incidental finding in apparently asymptomatic subjects): - Regional wall motion or global systolic/diastolic dysfunction, with or without ventricular dilatation, with or without increased wall thickness, with or without pericardial effusion, with or without endocavitary thrombi   **IV. Tissue characterization by CMR**   - Oedema and/or LGE of classical myocarditic pattern |
| **Definition** |
| Clinically suspected myocarditis if **≥1 clinical presentation** and **≥1 diagnostic criterion** from different categories, in the absence of:  (1) Angiographically detectable coronary artery disease (coronary stenosis ≥50%);  (2) Known pre-existing cardiovascular disease or extra-cardiac causes explaining the syndrome (e.g. valve disease, congenital heart disease, hyperthyroidism, etc.).  *For asymptomatic patients: ≥2 diagnostic criteria should be met.* |

**Supplementary Table 4**. **Updated Lake Louise Criteria for the CMR-based Diagnosis of Myocarditis**

Adapted from Ferreira et al. [13]

| CMR Diagnostic Domain | Specific Markers | Definition / Threshold |
| --- | --- | --- |
| T1-based markers of nonischemic myocardial injury | 1. Abnormal native T1 mapping  2. Increased ECV  3. LGE with a nonischemic distribution pattern (typically subepicardial or mid-wall) | Abnormal values defined relative to local reference range or control population |
| T2-based markers of myocardial edema | 1. Abnormal T2 mapping  2. Regional or global high signal intensity on T2-weighted imaging | Abnormal values or visually increased myocardial signal intensity (edema) |
| Diagnosis of myocarditis requires: ≥1 positive T1-based marker and ≥1 positive T2-based marker | | |
| Supportive findings: Pericardial effusion, pericardial enhancement, or LV systolic dysfunction may increase diagnostic confidence but are not mandatory | | |

**Legend**. ECV: extracellular volume; LGE: late gadolinium enhancement.

**Supplementary Table 5.** **Evaluation of methodological quality of the included case reports and case series, evaluated according to the Murad tool** [19]

| **Study** | **Item#1** | **#2** | **#3** | **#4** | **#5** | **#6** | **#7** | **#8** | **Score** |
| --- | --- | --- | --- | --- | --- | --- | --- | --- | --- |
| Adachi et al [21] | 0 | 1 | 1 | 1 | 1 | 0 | 1 | 1 | 6 |
| Belfeki et al [22] | 1 | 1 | 1 | 1 | 0 | 0 | 1 | 1 | 6 |
| Brick et al [32] | 1 | 1 | 1 | 1 | 1 | 0 | 0 | 1 | 6 |
| Buchanan et al [23] | 1 | 1 | 1 | 1 | 0 | 0 | 1 | 1 | 6 |
| Colantuono et al [24] | 1 | 1 | 1 | 1 | 1 | 1 | 1 | 1 | 8 |
| Goyack et al [25] | 1 | 1 | 1 | 1 | 0 | 0 | 1 | 1 | 6 |
| Higashitani et al [26] | 1 | 1 | 1 | 1 | 1 | 0 | 1 | 1 | 7 |
| Huynh et al [27] | 1 | 1 | 1 | 1 | 1 | 0 | 1 | 1 | 7 |
| Inglis et al [28] | 1 | 1 | 1 | 1 | 1 | 0 | 1 | 1 | 7 |
| Kodaka et al [29] | 1 | 1 | 1 | 1 | 0 | 0 | 1 | 1 | 6 |
| Panina et al [30] | 1 | 1 | 1 | 1 | 0 | 0 | 1 | 1 | 6 |
| Rao et al [31] | 1 | 1 | 1 | 1 | 1 | 0 | 0 | 1 | 6 |
| Song et al [35] | 1 | 1 | 1 | 1 | 0 | 0 | 1 | 1 | 6 |
| Trovato et al, patient #1 [36] | 1 | 1 | 1 | 1 | 0 | 0 | 1 | 1 | 6 |
| Trovato et al, patient #2 [36] | 1 | 1 | 1 | 1 | 0 | 1 | 0 | 1 | 6 |
| Trovato et al, patient #3 [36] | 1 | 1 | 1 | 1 | 0 | 1 | 1 | 1 | 7 |
| Truong et al [34] | 1 | 1 | 1 | 1 | 0 | 1 | 0 | 1 | 6 |
| Ulu et al [33] | 1 | 1 | 1 | 1 | 0 | 0 | 1 | 1 | 6 |

*Murad tool items:*

1. (*Selection*) Does the patient(s) represent(s) the whole experience of the investigator (centre) or is the selection method unclear to the extent that other patients with similar presentation may not have been reported?
2. (*Ascertainment*) Was the exposure adequately ascertained?
3. (*Ascertainment*) Was the outcome adequately ascertained?
4. (*Causality*) Were other alternative causes that may explain the observation ruled out?
5. (*Causality*) Was there a challenge/rechallenge phenomenon?
6. (*Causality*) Was there a dose–response effect?
7. (*Causality*) Was follow-up long enough for outcomes to occur?
8. (*Reporting*) Is the case(s) described with sufficient details to allow other investigators to replicate the research or to allow practitioners make inferences related to their own practice?

**Supplementary Table 6**. Comprehensive clinical characteristics of patients treated with anti-IL5/5R or anti-IL4/13 monoclonal antibodies from the contemporary cohort.

| **Patient** | **Gender (F/M), age (y)** | **BP or CS myocarditis** | **Indications to anti-IL5/5R or anti-IL4/13 mAb therapy** | **Clinical presentation, NYHA class** | **TnI elevations (yes/no), absolute value (ng/L)** | **Elevated eosinophils (yes/no)** | **Echo LVEF at diagnosis (%)** | **Intraventricular thrombus (yes/no)** | **Type of monoclonal antibody (mepolzumab, benralizumab, dupilumab)** | **Glucocorticoid at diagnosis (yes/no)** | **Duration of follow-up (months)** | **NYHA at follow-up** | **TnI elevation, eosinophils elevation at follow-up (yes/no)** | **LVEF at follow-up (%)** | **GC at follow-up (yes/no)** | **Relapse, Htx, death** |
| --- | --- | --- | --- | --- | --- | --- | --- | --- | --- | --- | --- | --- | --- | --- | --- | --- |
| Patient 1 | F, 37 | CS | EGPA (uncontrolled SEA and CRSwNP) | Infarct-like, I | Yes (10182.0) | Yes | 52 | Yes | Mepo | Yes | 30.0 | I | No | 70 | No | No |
| Patient 2 | M, 41 | CS | EGPA (Remission maintenance, steroid-sparing) | Infarct-like, I | Yes (3417.0) | Yes | 52 | No | Benra | Yes | 97.4 | I | Yes, Eos | 54 | No | No |
| Patient 3 | F, 27 | CS | EGPA (uncontrolled SEA) | Infarct-like, I | Yes (19000.0) | Yes | 48 | Yes | Mepo | Yes | 134.6 | I | No | 44 | No | No |
| Patient 4 | F, 53 | BP | EGPA (Remission maintenance) | Heart Failure, II | Yes (4270.0) | No | 35 | No | Mepo | No | 37.0 | I | Yes, TnI (23.0) | 51 | No | No |
| Patient 5 | M, 46 | BP | Uncontrolled SEA | Heart Failure, III | No (nan) | Yes | 23 | No | Dupi | Yes | 120.4 | II | Yes, Eos | 38 | No | No |
| Patient 6 | M, 42 | CS | EGPA (Remission maintenance) | Heart Failure, III | Yes (4955.0) | Yes | 42 | No | Mepo | Yes | 5.0 | I | No | 57 | No | No |
| Patient 7 | M, 42 | BP | Uncontrolled SEA and CRSwNP | Infarct-like, II | Yes (3473.0) | Yes | 60 | Yes | Mepo | Yes | 44.1 | I | No | 56 | No | No |
| Patient 8 | M, 61 | CS | HES (Remission induction) | Heart Failure, III | Yes (6838.0) | Yes | 54 | No | Mepo | Yes | 5.0 | I | No | 54 | Yes | No |
| Patient 9 | F, 33 | BP | HES (Remission induction) | Infarct-like, I | Yes (5229.0) | Yes | 31 | Yes | Mepo | Yes | 7.0 | I | No | 62 | No | No |
| Patient 10 | M, 60 | CS | EGPA (Remission maintenance) | Heart Failure, II | Yes (3713.0) | Yes | 44 | No | Mepo | Yes | 32.0 | I | No | 61 | No | No |
| Patient 11 | M, 30 | CS | EGPA (Uncontrolled SEA) | Infarct-like, I | Yes (5000.0) | Yes | 61 | No | Benra | Yes | 69.2 | I | No | 61 | No | No |
| Patient 12 | F, 77 | BP | EGPA (Uncontrolled SEA) | Infarct-like, I | Yes (nan) | Yes | 46 | No | Mepo | Yes | 61.0 | II | No | 55 | No | No |
| Patient 13 | M, 83 | BP | EGPA (Remission Induction) | Infarct-like, I | Yes (360.0) | Yes | 40 | Yes | Mepo | Yes | 35.2 | I | No | 52 | No | No |
| Patient 14 | M, 49 | CS | EGPA (Remission Induction) | Infarct-like, I | Yes (552.0) | Yes | 60 | No | Mepo | Yes | 28.0 | I | Yes, TnI (21.0) | 59 | No | No |
| Patient 15 | M, 47 | CS | EGPA (Remission maintenance) | Infarct-like, I | Yes (806.0) | Yes | 44 | No | Mepo | Yes | 108.0 | I | No | 61 | No | No |
| Patient 16 | F, 55 | CS | EGPA (Remission Induction) | Infarct-like, I | Yes (287.0) | Yes | 64 | Yes | Mepo | Yes | 23.0 | I | No | nan | No | No |
| Patient 17 | M, 71 | CS | EGPA (Remission Induction) | Infarct-like, I | Yes (1150.0) | Yes | 45 | No | Mepo | Yes | 18.0 | II | No | 54 | Yes | No |
| Patient 18 | M, 57 | CS | EGPA (Uncontrolled CRSwNP) | Infarct-like, I | Yes (733.0) | Yes | 65 | No | Mepo | Yes | 47.0 | I | No | 52 | No | No |
| Patient 19 | F, 39 | CS | EGPA (Uncontrolled CRSwNP; Remission maintenance) | Infarct-like, I | Yes (1367.0) | Yes | 50 | No | Mepo | Yes | 51.0 | I | No | 60 | No | No |

**Legend**: Benra: Benralizumab; BP: Biopsy-proven; CRSwNP: Chronic Rhinosinusitis with Nasal Polyps; CS: Clinically Suspected; Dupi: Dupilumab; EGPA: Eosinophilic Granulomatosis with Polyangiitis; Eos: Eosinophils; GC: Glucocorticoids; HES: Hypereosinophilic Syndrome; Htx: Heart transplantation; LVEF: Left Ventricular Ejection Fraction; mAb: Monoclonal Antibody; Mepo: Mepolizumab; NYHA: New York Heart Association; SEA: Severe Eosinophilic Asthma; TnI: Troponin I

**Supplementary Table 7. Additional Immunomodulatory and Immunosuppressive Regimens Across Subgroups**

| **Therapy category** | **Total (N=17)** | **Padova (N=12)** | **Literature (N=5)** |
| --- | --- | --- | --- |
|  |  |  |  |
| Azathioprine | 2 (12%) | 2 (17%) | 0 |
| Methotrexate | 1 (6%) | 1 (8%) | 0 |
| Cyclophosphamide | 2 (12%) | 1(8%) | 1 (20%) |
| Rituximab | 6 (36%) | 4 (33%) | 2 (40%) |
| Combination regimens* | 6 (36%) | 4 (33%) | 2 (40%) |

**Legend:** *Combination regimens include IVIG administered together with another immunosuppressive agent, as well as combinations of two additional IS/immunomodulatory therapies. Percentages (%) are calculated on the total number of patients who received other immunosuppressive/immunomodulatory regimens at diagnosis.

**Supplementary Table 8** Comparative clinical, diagnostic, and therapeutic features at diagnosis of patients with biopsy-proven eosinophilic myocarditis (n=11) versus those with non-eosinophilic biopsy-proven myocarditis under steroid therapy or with clinically suspected myocarditis (n=26), within a cohort of 37 patients treated with anti-IL-5/5R or anti-IL-4/13 monoclonal antibodies.

| **Characteristics** | **N** | **Biopsy-proven EM**  **N = 11** | **Clinically-suspected EM**  **N = 26** | **p-value^1^** |
| --- | --- | --- | --- | --- |
| **Gender** (male) | 37 | 7 (64%) | 12 (46%) | 0.33 |
| **Age at diagnosis** | 37 | 46 (27, 60) | 48 (39, 59) | 0.84 |
| **EGPA/HES diagnosis** | 37 |  |  | **0.030** |
| EGPA |  | 4 (36%) | 20 (77%) |  |
| HES |  | 1 (9%) | 3 (12%) |  |
| Eosinophilic asthma |  | 3 (27%) | 2 (8%) |  |
| **Cardiac symptoms at diagnosis** | 35 | 10 (100%) | 21 (84%) | 0.30 |
| **Family history of autoimmune diseases** | 18 | 0 (0%) | 2 (12%) | >0.99 |
| **Personal history of autoimmune disease** | 37 | 2 (18%) | 4 (15%) | >0.99 |
| **Allergy** | 23 | 3 (100%) | 13 (65%) | 0.53 |
| **Asthma** | 37 | 7 (64%) | 24 (92%) | 0.051 |
| **Smoking history** | 16 | 0 (0%) | 8 (57%) | 0.47 |
| **BVASv3 at diagnosis** | 22 | 17.5 (13.0, 21.5) | 15.5 (12.2, 19.5) | 0.90 |
| **Multisystem Involvement at the Time of EGPA/HES Diagnosis** | 24 |  |  |  |
| EGPA_HES_Constitutional_symptoms | 24 | 2 (50%) | 12 (60%) | >0.99 |
| EGPA_HES_Cutaneous_symptoms | 24 | 1 (25%) | 6 (30%) | >0.99 |
| EGPA_HES_ENT_symptoms | 24 | 2 (50%) | 15 (75%) | 0.55 |
| EGPA_HES_Pulmonary_symptoms | 24 | 4 (100%) | 20 (100%) |  |
| EGPA_HES_Cardiac_symptoms | 24 | 4 (100%) | 16 (80%) | >0.99 |
| EGPA_HES_Digestive_symptoms | 24 | 1 (25%) | 3 (15%) | 0.54 |
| EGPA_HES_Renal_symptoms | 24 | 0 | 1 (5.0%) | >0.99 |
| EGPA_HES_Neurological_symptoms | 24 | 2 (50%) | 6 (30%) | 0.58 |
| **Clinical presentation** | 37 |  |  | **0.044** |
| Heart Failure |  | 9 (82%) | 9 (35%) |  |
| Infarct-like |  | 2 (18%) | 14 (54%) |  |
| Asymptomatic |  | 0 (0%) | 3 (12%) |  |
| **NYHA II-III at diagnosis** | 28 | 4 (80%) | 10 (43%) | 0.28 |
| **Fulminant presentation** | 37 | 3 (27%) | 4 (15%) | 0.40 |
| **Laboratory biomarkers at diagnosis** |  |  |  |  |
| TnI elevation | 36 | 6 (55%) | 24 (92%) | **0.006** |
| TnI level (ng/l) | 26 | 2,001 (1,046, 3,250) | 3,417 (806, 5,229) | 0.75 |
| CRP elevation | 36 | 5 (45%) | 17 (68%) | 0.27 |
| CRP level (mg/l) | 21 | 48 (8, 63) | 33 (15, 95) | 0.82 |
| NTproBNP elevation | 32 | 6 (55%) | 17 (81%) | 0.21 |
| NTproBNP level (pg/ml) | 19 | 1,585 (640, 1,723) | 1,416 (858, 6,509) | 0.82 |
| Eosinophils elevation | 37 | 11 (100%) | 25 (96%) | >0.99 |
| Eosinophil count (×10⁹/L) | 33 | 5 (3, 9) | 5 (2, 9) | 0.72 |
| **Autoantibodies profile at diagnosis** |  |  |  |  |
| AHA positivity | 13 | 1 (50%) | 5 (45%) | >0.99 |
| AIDA positivity | 13 | 0 (0%) | 4 (36%) | >0.99 |
| ANCA positivity | 23 | 0 (0%) | 1 (5.6%) | >0.99 |
| FR positivity | 11 | 0 (0%) | 6 (60%) | 0.45 |
| **Abnormal ECG findings at diagnosis*** | 21 | 2 (100%) | 12 (60%) | 0.52 |
| **Echocardiography at diagnosis** |  |  |  |  |
| Reduced LVEF | 37 | 10 (91%) | 17 (65%) | 0.22 |
| Echo LVEF (%) | 21 | 40 (40, 50) | 48 (43, 55) | 0.53 |
| Echo LVEDi (ml/m2) | 16 | 80 (73, 86) | 67 (54, 75) | 0.38 |
| Echo FAC (%) | 16 | 36 (35, 36) | 44 (38, 51) | 0.20 |
| Echo RVED area (mm2) | 14 | 24.0 (23.5, 24.5) | 17.5 (16.8, 22.0) | 0.27 |
| Moderate/severe mitral insufficiency | 18 | 1 (50%) | 5 (31%) | >0.99 |
| Pericardial effusion | 37 | 5 (45%) | 8 (31%) | 0.46 |
| Endocavitary thrombus | 37 | 2 (18%) | 6 (23%) | >0.99 |
| **CMR at diagnosis** | 37 | 8 (73%) | 24 (92%) | 0.14 |
| Edema on CMR | 15 | 1 (100%) | 10 (71%) | >0.99 |
| LGE on CMR | 33 | 8 (89%) | 24 (100%) | 0.27 |
| LGE pattern | 32 |  |  | 0.59 |
| Subendocardial (ischemic pattern) |  | 7 (88%) | 22 (92%) |  |
| Epicardial/intramyocardial spots |  | 1 (12%) | 2 (8%) |  |
| **Treatment** |  |  |  |  |
| **Type of mAb** | 37 |  |  | >0.99 |
| Mepolizumab |  | 9 (82%) | 20 (77%) |  |
| Benralizumab |  | 2 (18%) | 4 (15%) |  |
| Dupilumab |  | 0 (0%) | 2 (7.7%) |  |
| **Time from myocarditis onset to initiation of mAb therapy** (months) | 36 | 0 (0, 2) | 8 (1, 34) | **0.007** |
| **GC use at diagnosis** | 35 | 10 (91%) | 23 (96%) | 0.54 |
| **IS or other immunomodulatory treatments at diagnosis** | 37 | 2 (18%) | 15 (58%) | 0.028 |
| **Genetic testing performed** | 19 | 2 (100%) | 8 (47%) | 0.47 |
| Genetic testing results (positive) | 10 | 2 (100%) | 8 (100%) |  |

Data are presented as n (%) andmedian (Q1, Q3). **Legend**: AHA, anti-heart autoantibodies; AIDA, anti-intercalated disk autoantibodies; ANCA, anti-neutrophil cytoplasmic antibodies; BNP, brain natriuretic peptide; BVAS, Birmingham Vasculitis Activity Score; CMR, cardiovascular magnetic resonance; CRP, C-reactive proteine; cTn, cardiac troponin; EGPA, eosinophilic granulomatosis with polyangiitis; EMB, endomyocardial biopsy; ENT, ear-nose-throat; FAC, fractional area change; GC, glucocorticoid; HES, Hypereosinophilic Syndrome; IS, immunosuppression; LGE, late gadolinium enhancement; LVEF, left ventricular ejection fraction; LVEDVi, left ventricular end-diastolic volume indexed; mAb, monoclonal antibody; NYHA, New York Heart Association; PCR, polymerase chain reaction; RF, rheumatoid factor; RVED, right ventricular end-diastolic; TAPSE, tricuspid annular plane systolic excursion.

*Abnormal ECG was defined as the presence of any repolarization (ST-T abnormalities), depolarization (pathological Q waves, low QRS voltage), or conduction disturbances (AV or bundle branch block) on ECG at diagnosis or at follow-up.

**Supplementary Table 9**. Comparative clinical, diagnostic, and therapeutic features at follow-up of patients with biopsy-proven eosinophilic myocarditis (n=11) versus those with non-eosinophilic biopsy-proven myocarditis under steroid therapy or with clinically suspected myocarditis (n=26), within a cohort of 37 patients treated with anti-IL-5/5R or anti-IL-4/13 monoclonal antibodies.

| **Characteristic** | **N** | **Clinically-suspected EM**  **N = 26** | **Biopsy-proven EM**  **N = 11** | **p-value** |
| --- | --- | --- | --- | --- |
| **Duration of follow-up** | 19 | 37 (23, 69) | 40 (37, 42) | >0.99 |
| **Myocarditis relapse** | 37 | 0 | 0 |  |
| **NYHA II-III at follow-up** | 19 | 3 (18%) | 0 (0%) | >0.99 |
| **Laboratory biomarkers at follow-up** |  |  |  |  |
| TnI elevation | 17 | 2 (13%) | 0 (0%) | >0.99 |
| PCR elevation | 19 | 0 | 0 |  |
| NTproBNP elevation | 18 | 8 (50%) | 1 (50%) | >0.99 |
| NTproBNP level (pg/ml) | 15 | 196 (102, 524) | 493 (493, 493) | 0.80 |
| Eosinophils elevation | 19 | 2 (12%) | 0 (0%) | >0.99 |
| **Autoantibodies profile at follow-up** |  |  |  |  |
| AHA positivity | 10 | 3 (38%) | 1 (50%) | >0.99 |
| AIDA positivity | 10 | 2 (25%) | 0 (0%) | >0.99 |
| ANCA positivity | 17 | 2 (13%) | 0 (0%) | >0.99 |
| **Abnormal ECG findings at follow-up *** | 16 | 7 (50%) | 1 (50%) | >0.99 |
| **Echocardiography at follow-up** |  |  |  |  |
| Reduced LVEF | 18 | 3 (19%) | 0 (0%) | >0.99 |
| Echo LVEF (%) | 19 | 56.0 (53.5, 61.0) | 56.0 (54.0, 58.0) | 0.87 |
| Echo LVEDi (ml/m2) | 17 | 62 (52, 66) | 70 (67, 73) | 0.37 |
| Echo FAC (%) | 15 | 50 (42, 57) | 45 (40, 50) | 0.55 |
| Echo RVED area (mm2) | 15 | 17.0 (15.0, 20.0) | 23.5 (22.8, 24.2) | 0.060 |
| Moderate/severe mitral insufficiency | 18 | 0 | 0 |  |
| Pericardial effusion | 19 | 0 | 0 |  |
| Endocavitary thrombus | 18 | 0 | 0 |  |
| **CMR at follow-up** | 19 | 9 (53%) | 1 (50%) | >0.99 |
| Edema on CMR | 10 | 0 | 0 |  |
| LGE on CMR | 10 | 8 (89%) | 1 (100%) | >0.99 |
| LGE pattern | 9 |  |  | 0.33 |
| Subendocardial (ischemic pattern) |  | 7 (83%) | 0 (0%) |  |
| Epicardial/intramyocardial spots |  | 1 (12%) | 1 (100%) |  |
| Reduced number of LGE-positive segments | 9 | 5 (62%) | 0 (0%) | 0.44 |
| **Therapy at follow-up** |  |  |  |  |
| mAb therapy withdrawn | 19 | 0 | 0 |  |
| Ongoing GC therapy | 21 | 3 (17%) | 1 (33%) | 0.49 |
| Ongoing IS/other immunomodulatory therapy | 19 | 1 (5.9%) | 0 (0%) | >0.99 |
| **Total duration of mAb therapy** (months) | 19 | 22 (13, 32) | 36 (33, 40) | 0.16 |
| **Duration of mAb therapy without concomitant GC or IS** (months) | 19 | 13 (5, 24) | 29 (26, 32) | 0.14 |

Data are presented as n (%) and; median (Q1, Q3). **Legend**: AHA, anti-heart autoantibodies; AIDA, anti-intercalated disk autoantibodies; ANCA, anti-neutrophil cytoplasmic antibodies; BNP, brain natriuretic peptide; BVAS, Birmingham Vasculitis Activity Score; CMR, cardiovascular magnetic resonance; CRP, C-reactive proteine; cTn, cardiac troponin; EGPA, eosinophilic granulomatosis with polyangiitis; EMB, endomyocardial biopsy; ENT, ear-nose-throat; FAC, fractional area change; GC, glucocorticoid; HES, Hypereosinophilic Syndrome; IS, immunosuppression; LGE, late gadolinium enhancement; LVEF, left ventricular ejection fraction; LVEDVi, left ventricular end-diastolic volume indexed; mAb, monoclonal antibody; NYHA, New York Heart Association; PCR, polymerase chain reaction; RF, rheumatoid factor; RVED, right ventricular end-diastolic; TAPSE, tricuspid annular plane systolic excursion.

*Abnormal ECG was defined as the presence of any repolarization (ST-T abnormalities), depolarization (pathological Q waves, low QRS voltage), or conduction disturbances (AV or bundle branch block) on ECG at diagnosis or at follow-up.

**Supplementary Table 10.** Comparative clinical, diagnostic, and therapeutic characteristics at baseline and follow-up in patients with EM treated with anti–IL-5/5R or anti–IL-4/13 mAb (n=37) versus non–mAb-treated patients with EM (n=12).

| **Characteristic** | **N** | **Yes**  **N = 37** | **No**  **N = 12** | **p-value** |
| --- | --- | --- | --- | --- |
| **Gender** (male) | 49 | 19 (51%) | 8 (67%) | 0.35 |
| **Age at diagnosis** | 49 | 47 (34, 60) | 32 (24, 64) | 0.41 |
| **EGPA/HES diagnosis** | 49 |  |  | **<0.001** |
| EGPA |  | 24 (65%) | 1 (8.3%) |  |
| HES |  | 6 (16%) | 1 (8.3%) |  |
| **Family history of autoimmune diseases** | 49 | 2 (11%) | 3 (25%) | 0.36 |
| **Personal history of autoimmune disease** | 49 | 6 (16%) | 5 (42%) | 0.11 |
| **Allergy** | 49 | 16 (70%) | 5 (42%) | 0.15 |
| **Asthma** | 49 | 31 (84%) | 1 (8.3%) | **<0.001** |
| **Clinical presentation** | 49 |  |  | 0.088 |
| Heart Failure |  | 18 (49%) | 4 (33%) |  |
| Pseudoinfarction |  | 16 (43%) | 6 (50%) |  |
| Arrhythmic |  | 0 (0%) | 2 (17%) |  |
| Asymptomatic |  | 3 (8.1%) | 0 (0%) |  |
| **NYHA II-III at diagnosis** | 40 | 14 (50%) | 6 (50%) | 0.90 |
| **Fulminant presentation** | 46 | 5 (14%) | 0 (0%) | 0.57 |
| **Laboratory biomarkers at diagnosis** |  | 32 (86%) | 9 (100%) |  |
| TnI elevation |  | 29 (81%) | 6 (67%) | 0.39 |
| TnI level (ng/l) |  | 3,261 (864, 5114) | 11,857 (2,547, 19,180) | 0.13 |
| CRP elevation | 46 | 22 (59%) | 7 (70%) | 0.72 |
| CRP level (mg/l) |  | 36 (14, 93) | 29 (16, 100) | 0.89 |
| NTproBNP elevation | 36 | 23 (70%) | 4 (100%) | 0.89 |
| NTproBNP level (pg/ml) | 22 | 1557 (725, 5568) | 5,292  (2,768, 8,836) | 0.59 |
| Eosinophils elevation | 43 | 36 (97%) | 6 (100%) | >0.99 |
| Eosinophil count (×10⁹/L) | 36 | 5 (2, 10) | 8 (5, 24) | 0.55 |
| **Autoantibodies profile at diagnosis** |  |  |  |  |
| AHA positivity | 21 | 6 (46%) | 4 (50%) | >0.99 |
| AIDA positivity | 20 | 4 (31%) | 3 (43%) | 0.65 |
| ANCA positivity | 28 | 1 (4%) | 0 (0%) | >0.99 |
| **Abnormal ECG findings at diagnosis*** | 27 | 14 (63%) | 5 (83%) | 0.63 |
| **Echocardiography at diagnosis** |  |  |  |  |
| Reduced LVEF | 49 | 27 (73%) | 5 (42%) | 0.080 |
| Echo LVEF (%) | 33 | 47 (41, 55) | 53 (44, 57) | 0.54 |
| Echo VTDi (ml/m2) | 24 | 68 (54, 77) | 56 (46, 80) | 0.50 |
| Moderate/severe mitral insufficiency | 30 | 6 (33%) | 4 (33%) | >0.99 |
| Pericardial effusion | 43 | 13 (35%) | 1 (17%) | 0.65 |
| Endocavitary thrombus | 44 | 8 (22%) | 3 (43%) | 0.34 |
| **CMR at diagnosis** | 49 | 32 (86%) | 7 (58%) | 0.050 |
| Edema on CMR | 21 | 11 (73%) | 6 (100%) | 0.28 |
| LGE on CMR | 40 | 32 (97%) | 7 (100%) | >0.99 |
| LGE pattern | 39 |  |  | **0.006** |
| Subendocardial (ischemic pattern) |  | 29 (91%) | 3 (43%) |  |
| Epicardial/intramyocardial spots |  | 3 (9.3%) | 4 (57%) |  |
| **Endomyocardial biopsy at diagnosis** | 49 | 19 (51%) | 12 (100%) | **0.002** |
| Histological type | 28 |  |  | 0.23 |
| Eosinophylic |  | 12 (75%) | 12 (100%) |  |
| Lymphocytic |  | 3 (19%) | 0 (0%) |  |
| Polymorph |  | 1 (6%) | 0 (0%) |  |
| Endomyocardial fibrosis | 27 | 4 (21%) | 3 (38%) | 0.63 |
| PCR on EMB | 31 | 19 (100%) | 10 (83%) | 0.14 |
| Negative PCR results | 29 | 19 (100%) | 7 (70%) | 0.033 |
| **Treatment** |  |  |  |  |
| GC use at diagnosis | 44 | 33 (94%) | 12 (100%) | >0.99 |
| Immunosuppression at diagnosis | 46 | 17 (46%) | 6 (67%) | 0.26 |
| **Duration of follow-up** | 31 | 37 (26, 65) | 24 (12, 69) | 0.43 |
| **Myocarditis Relapse** | 49 | 0 | 3 (25%) | **0.012** |
| **NYHA II-III at follow-up** | 31 | 3 (16%) | 1 (8.3%) | 0.14 |
| **Laboratory biomarkers at follow-up** |  |  |  |  |
| TnI elevation | 24 | 2 (12%) | 3 (43%) | 0.13 |
| NTproBNP elevation | 25 | 9 (50%) | 3 (43%) | >0.99 |
| Eosinophils elevation | 25 | 2 (11%) | 4 (67%) | **0.015** |
| Eosinophil count (×10⁹/L) | 21 | 0.04 (0.03, 0.08) | 0.85 (0.74, 0.95) | **0.043** |
| **Abnormal ECG findings at follow-up *** | 23 | 8 (50%) | 5 (71%) | 0.41 |
| **Echocardiography at follow-up** |  |  |  |  |
| Reduced LVEF | 30 | 3 (17%) | 4 (33%) | 0.39 |
| Echo LVEF (%) | 31 | 56 (53, 60) | 52 (48, 55) | 0.092 |
| Echo VTDi (ml/m2) | 29 | 63 (52, 67) | 74 (64, 75) | 0.10 |
| Echo TAPSE (mm) | 21 | 20.0 (18.0, 26.0) | 22.0 (19.8, 22.8) | 0.88 |
| Echo FAC (%) | 25 | 50 (42, 56) | 41 (36, 44) | 0.055 |
| Echo RVED area (mm2) | 26 | 20.0 (15.0, 21.5) | 17.0 (16.0, 22.0) | 0.88 |
| **Therapy at follow-up** |  |  |  |  |
| Ongoing GC therapy | 26 | 4 (19%) | 2 (40%) | 0.56 |
| Ongoing IS/other immunomodulatory therapy | 31 | 1 (5.3%) | 2 (17%) | 0.54 |

Data are presented as n (%) and; median (Q1, Q3). **Legend**: AHA, anti-heart autoantibodies; AIDA, anti-intercalated disk autoantibodies; ANCA, anti-neutrophil cytoplasmic antibodies; BNP, brain natriuretic peptide; BVAS, Birmingham Vasculitis Activity Score; CMR, cardiovascular magnetic resonance; CRP, C-reactive proteine; cTn, cardiac troponin; EGPA, eosinophilic granulomatosis with polyangiitis; EMB, endomyocardial biopsy; ENT, ear-nose-throat; FAC, fractional area change; GC, glucocorticoid; HES, Hypereosinophilic Syndrome; IS, immunosuppression; LGE, late gadolinium enhancement; LVEF, left ventricular ejection fraction; LVEDVi, left ventricular end-diastolic volume indexed; mAb, monoclonal antibody; NYHA, New York Heart Association; PCR, polymerase chain reaction; RF, rheumatoid factor; RVED, right ventricular end-diastolic; TAPSE, tricuspid annular plane systolic excursion.

*Abnormal ECG was defined as the presence of any repolarization (ST-T abnormalities), depolarization (pathological Q waves, low QRS voltage), or conduction disturbances (AV or bundle branch block) on ECG at diagnosis or at follow-up.
